# A Lignin-Based Carbon Anode with Long-Cycle Stability for Li-Ion Batteries

**DOI:** 10.3390/ijms24010284

**Published:** 2022-12-23

**Authors:** Shiyue Li, Wenbin Luo, Qi He, Jie Lu, Jian Du, Yehan Tao, Yi Cheng, Haisong Wang

**Affiliations:** School of Light Industry and Chemical Engineering, Dalian Polytechnic University, Dalian 116034, China

**Keywords:** lignin, lithium-ion battery, anode, long-cycle performance

## Abstract

Due to its wide source and low cost, biomass-based hard carbon is considered a valuable anode for lithium-ion batteries (LIBs). Lignins, as the second most abundant source in nature, are being intensively studied as candidate anode materials for next generation LIBs. However, direct carbonization of pure lignin usually leads to low specific surface area and porosity. In this paper, we design a porous carbon material from natural lignin assisted by sacrificing a metal–organic framework (MOF) as the template. The MOF nanoparticles can disperse the lignin particles uniformly and form abundant mesopores in the composites to offer fast transfer channels for Li^+^. The as-prepared carbon anode shows a high specific capacity of 420 mAh g^−1^ with the capacity retention of 99% after 300 cycles at 0.2 A g^−1^. Additionally, it keeps the capacity retention of 85% after long cycle of 1000 cycles, indicating the good application value of the designed anode in LIBs. The work provides a renewable and low-cost candidate anode and a feasible design strategy of the anode materials for LIBs.

## 1. Introduction

Lithium-ion batteries (LIBs) are considered one of the most suitable battery systems for energy storage due to their cycle stability, high energy density and low self-discharge rate [1,2,3,4]. With the rapid development of the electronic vehicles, people have put forward an increasing demand for the LIBs with low cost and high performance. The cost and performance of LIBs is largely determined by the anode materials [5]. Graphene is a commercial anode material which is most used in LIBs, but it has high cost and relatively low theoretical specific capacity of 372 mAh g^−1^ [6,7]. Additionally, graphene is also prone to form lithium dendrites on the surface when embedded into more lithium ions, which has a serious impact on the safety and cycling performance of the batteries. Therefore, there is a necessity to develop a kind of anode material with low cost, high specific capacity and high safety [8].

Recently, biomass, which has the advantages of abundance, low cost, high carbon content and unique 3D structure, has aroused much attention [9]. The most abundant biomass in nature is the lignocellulosic materials that consist of cellulose, semi-cellulose and lignin. Among them, lignin has a unique aromatic carbon ring structure and abundant functional groups, which could allow for uniform pore formation and provide more active sites for the electrochemical reaction. This makes lignin a good precursor for carbon materials [9,10,11]. However, direct carbonization of the pure lignin usually leads to low specific surface area and more microporous structures, which is not helpful for the penetration of the electrolyte and fast transportation of Li^+^. 

Metal–organic frameworks (MOFs) have been explored to design 3D hierarchical structures owing to their high surface area and controlled channels [12,13]. It is also known that the N chemical bonds (N=C, N-C and N-H) in the organic ligand (2-methylimidazole) [14] can produce different kinds of N, or some defects in situ under high-temperature calcination [15,16,17,18,19]. Inspired by this, we designed a porous carbon material from natural lignin assisted by sacrificing a MOF as the template. The MOF nanoparticles can disperse the lignin particles uniformly and form abundant mesopores in the composites to offer fast transfer channels for Li^+^. The lignin serves as the 3D carbon framework after high-temperature calcination to provide high electronic conductivity for the composites. With the synergistic effect of the two kind of carbon materials with different microstructures, the final carbon composites show good mesoporous structure with high surface area and porosity, high conductivity of the electrons and fast transfer rate of Li^+^, thus leading to good rate performance and cycle performance as the anode for LIBs. 

## 2. Results and Discussion

**Figure 1:**
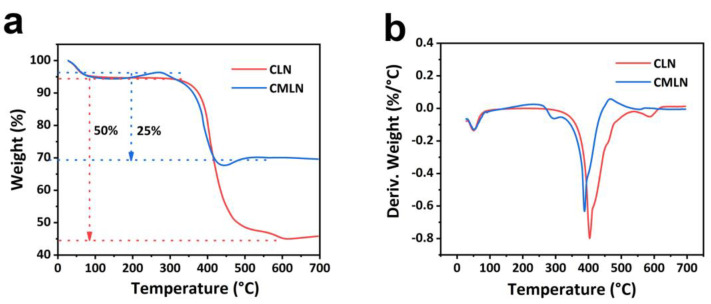
(**a**) TGA curves of CLN and CMLN; (**b**) DTG of CLN and CMLN.

To measure the content of the carbon in the samples, TGA was conducted. As shown in Figure 1a, the weight reduction at temperatures from 50 °C to 300 °C corresponds to the loss of water in CMLN and CLN, respectively [20]. Additionally, the sharp weight loss from about 300 °C is corresponding to the combustion of carbon in CMLN and CLN. The DTG curves of the two samples as shown in Figure 1b exhibit a sharp change from 300 °C to 400 °C, which reflect the combustion of the carbon in the composites. The results show that the carbon content of CMLN is 25% and the CLN shows a carbon content of 50%. The lower carbon content of the CMLN composites than the pure CLN is due to the addition of the MOF, which has a lower carbon content than the lignin.

**Figure 2:**
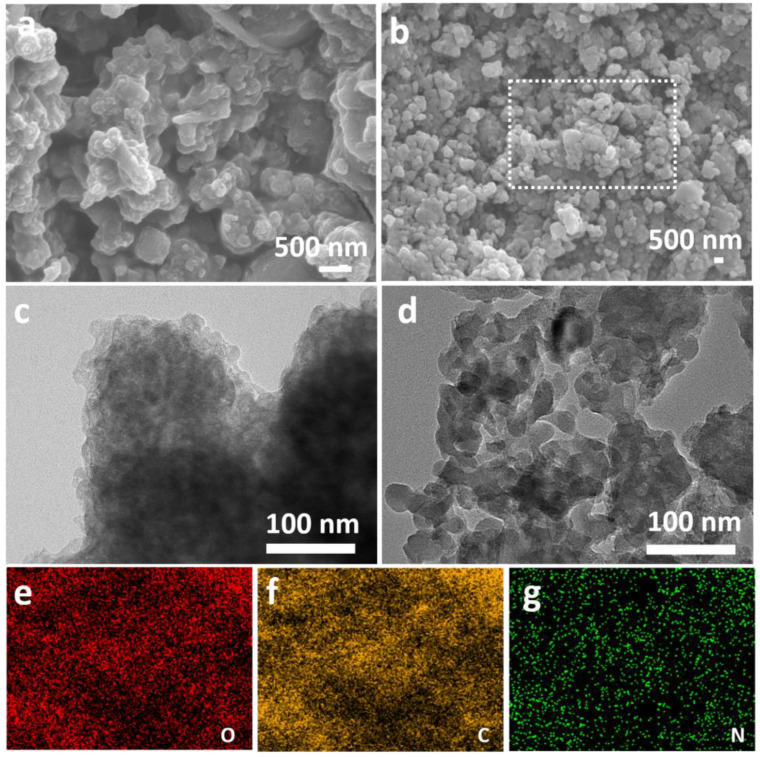
(**a**,**b**) SEM images of CLN and CMLN; (**c**,**d**) TEM images of CLN and CMLN; (**e**–**g**) elemental mapping of C, N and O for CMLN.

The morphologies of CLN and CMLN are shown in Figure 2a,b. It can be seen that the CLN particles are partially aggregated in a larger size. In contrast, the synthesized CMLN are distributed uniformly at a smaller size. The TEM images show that the carbon in CLN is aggregated into bulk (Figure 2c), but the carbon nanoparticles in CMLN composites exhibit sizes in the range of 20–200 nm and there are abundant mesopores distributed between the particles. (Figure 2d). The nano size of the CMLN can effectively shorten the transfer pathway for electrons, and the abundant mesopores are beneficial for the penetration of the electrolyte, thus promoting the Li^+^ transportation. The element mapping of the dashed area in Figure 2b displays that the main elements in CMLN composites are C, N and O, which are uniformly distributed in the CMLN composites as shown in Figure 2e,g. It should be mentioned that the N element is derived from the MOF, which is good for the further improvement of the electronic conductivity of the carbon anode [21]. 

**Figure 3:**
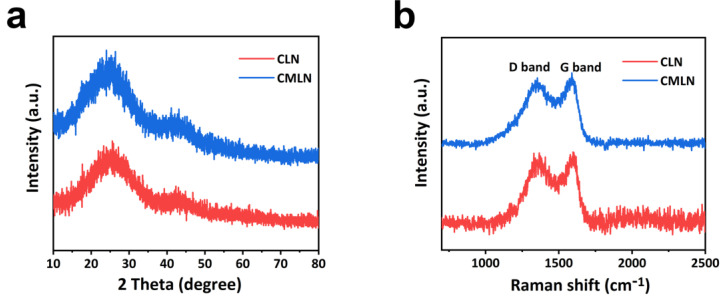
(**a**) XRD patterns of CLN and CMLN; (**b**) the Raman spectroscopy of CLN and CMLN.

The XRD patterns of the CLN and CMLN are displayed in Figure 3a. We see that two broad peaks centered at ∼25° and ∼44° are attributed to the (002) and (101), respectively, indicating the main property of the amorphous carbon in the two samples [22]. Raman spectra were conducted to further confirm the property of the carbon in the composites (Figure 3b). It can be seen that there are two obvious bands at 1342 and 1591 cm^−1^ in CLN and CMLN composites which are attributed to the disordered carbon (D band) and the ordered graphitic carbon (G band), respectively. Typically, the D band is due to structural defects caused by O, S and N doping, or chemical activation, and the G band originates from sp^2^ hybridization of carbons. It was known that the Raman integrated areas ratio (I_D_/I_G_) can evaluate the imperfect and disordered framework of porous carbon [23,24,25,26]. After analysis, the I_D_/I_G_ of CMLN is 2.805, which is larger than that of CLN (2.131), suggesting the more defect sites in CMLN. The more defect sites in CMLN are due to the N atom doping derived from the MOF, which are favorable for the enhancement of specific capacity.

**Figure 4:**
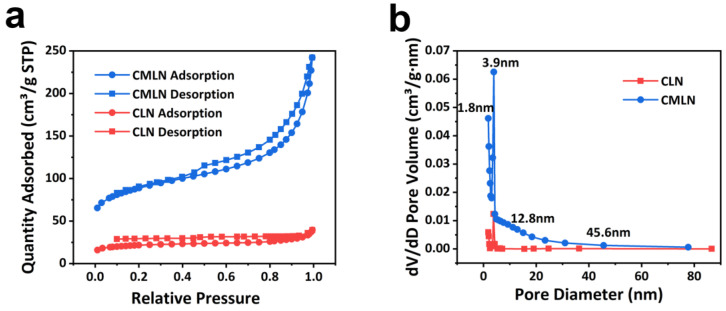
(**a**) N_2_ adsorption/desorption isotherm of CLN and CMLN; (**b**) pore distribution in CLN and CMLN.

N_2_ adsorption and desorption isotherms of two samples are given in Figure 4 to characterize surface area and porosity. The curves of CLN showed type I isotherms, implying the presence of little micropores (Figure 4a). However, curves of the CMLN show type IV curves with obvious hysteresis loop, indicating the existence of abundant mesopores [27]. The adsorption and desorption branches of this hysteresis loop are almost horizontal and correspond to the adsorption in narrow wedge-shaped pores [28]. The specific surface area of CMLN is 288.7766 cm^2^ g^−1^, which is much higher than that of CLN (68.4693 cm^2^ g^−1^). The distribution of pore sizes as shown in Figure 4b indicated that the pores in CMLN composites are mainly mesopores with the size of 3.9 nm, 12.8 nm and 45.6 nm. However, the CLN only displays little micropore distribution. The rich mesopores in CMLN composites can promote the penetration of the electrolyte and provide a large amount of transfer channels for Li^+^ ions. The large surface area can provide more active reaction sites for Li^+^ ions to increase specific capacity of the battery [29].

**Figure 5:**
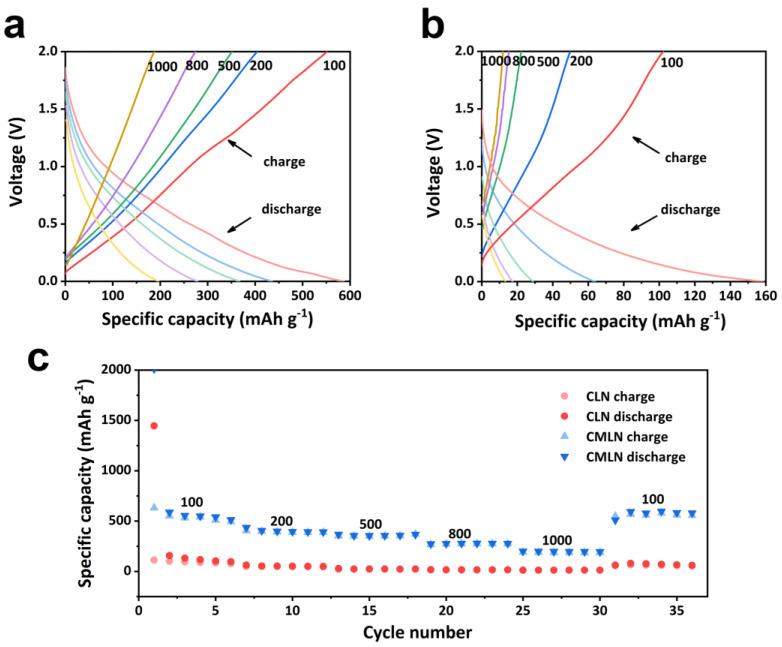
(**a**) Charge/discharge curves of CMLN at different current densities; (**b**) charge/discharge curves of CLN at different current densities; (**c**) the rate performance of CLN and CMLN with different current densities.

The charge/discharge profiles curves for the CLN and CMLN samples in the working voltage of 0.01~2 V are shown in Figure 5a,b, which exhibit the typical charge–discharge characteristics of amorphous carbon materials without obvious charge/discharge platforms [30]. The rate performance of CMLN and CLN under different current densities are presented in Figure 5c. It can be seen that the CMLN displays the specific capacities of 560, 420, 350, 270 and 180 m Ah g^−1^ under the current density of 100, 200, 500, 800 and 1000 mA g^−1^, respectively. With the current density decreased to 100 mA g^−1^, it can recover to 560 mAh g^−1^ again, which indicates the good electrochemical reversibility of the CMLN anode. However, the CLN anode only displays the specific capacity near to 0 mAh g^−1^ when the current density was higher than 500 mA g^−1^, suggesting the poor high-rate performance. The better rate performance of the CMLN can be attributed to two reasons: firstly, the abundant mesoporous structure and high porosity could promote the transportation of Li^+^ in the composites, which can match the fast electrochemical reaction; secondly, the carbon with N doping enhanced the electronic conductivity of the composites, which makes sure of fast electron transfer. 

**Figure 6:**
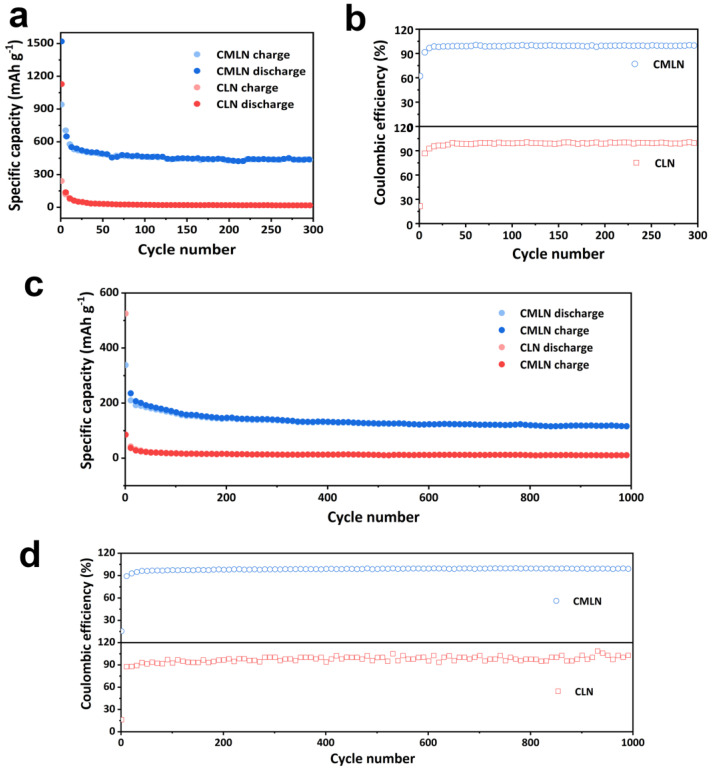
(**a**,**b**) Cycling performances and coulombic efficiency of CLN and CLMN at current density of 100 mA g^−1^; (**c**,**d**) cycling performances and Coulombic efficiency of CLN and CLMN at current density of 1000 mA g^−1^.

The cycling stability is another important performance for anodes. As we can observe from Figure 6a, the discharge specific capacity of CMLN still remains 420 mAh g^−1^ with a capacity retention of 99% after 300 cycles and the Coulombic efficient is close to 100% (Figure 6b), which suggested the good cycle stability of the CMLN anode. However, for CLN, it only displays a capacity retention of 30% after 300 cycles. To further explore the cycling stability of the CMLN anode, the long-term cycle performance was investigated as shown in Figure 6c,d. At the high current density of 1000 mA g^−1^, the CMLN anode still displayed 150 mAh g^−1^ with the capacity retention of 85% (start with the second cycle) after 1000 cycles, suggesting the excellent long-cycle stability of the CMLN anode. The good cycle performance of CMLN anode can be attributed to the good porous structure could reduce the volume expansion during the repeated charge–discharge process. The electrochemical performance comparison of the CMLN anode with other anodes reported in previous literature is listed in Table 1. We can see that the CMLN anode exhibited superiority, which indicated its good application value in LIBs. 

**Figure 7:**
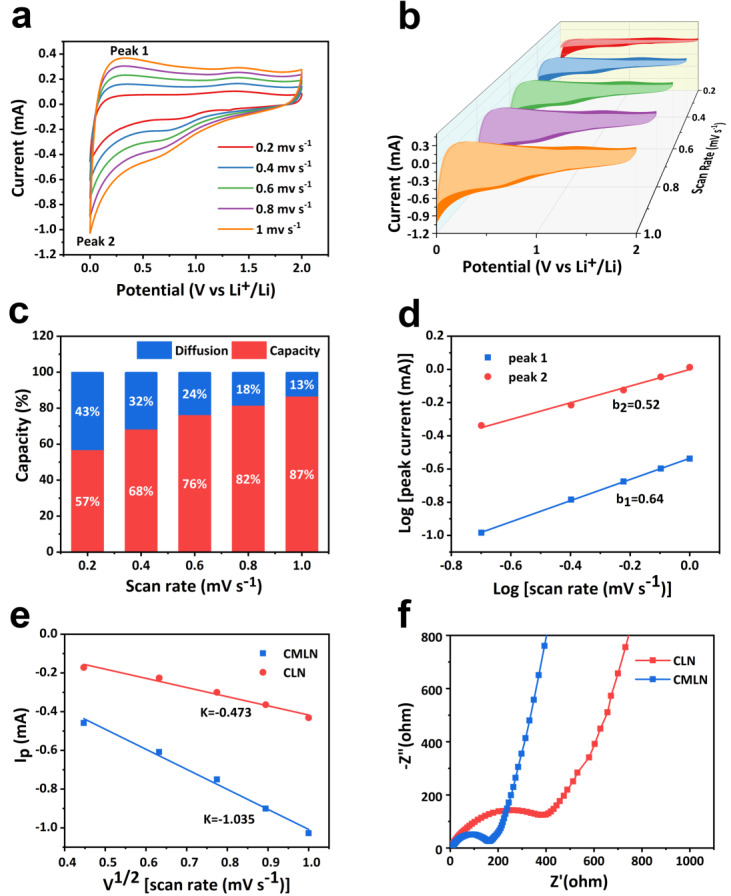
(**a**) CV curves of CMLN at different scan rates from 0.2 to 1 mV s^−1^; (**b**) fitting areas of the CV curves for CMLN with scan rates from 0.2 to 1 mV s^−1^; (**c**) capacitive and diffusion-controlled capacity contributions for the CMLN electrode at different scan rates; (**d**) relationship between the peak current and scan rate in the CV curve of CMLN; (**e**) linear fitting of CMLN and CLN based on peak 2 in the CV curves; (**f**) EIS of the batteries with CMLN and CLN anodes.

For the purpose of explaining the good electrochemical performance of the CMLN, the kinetic process in the electrode was investigated. We analyzed the CV curves of the batteries with the CMLN anode and CLN anode at different scan rates (Figure 7a and Figure S1). It can be seen that the CV curves of the two anodes both displayed no sharp peaks which were consistent with the charge–discharge curves (no obvious platforms). The storage mechanisms of Li^+^ in the electrodes mainly include the capacitive effect and the intercalation behavior based on diffusion, which could be presented as following equations [41]:

(1) $$i\left(v\right)=av^{b}$$

(2) $$Log\left(i\right)=Log\left(a\right)+bLog\left(v\right)$$

where *i* stands for the peak current, *ν* represents the sweep rate, *b* is the slope of the line as a function of *log* (*i*) and *log* (*v*) and *a* is the constant. If the *b* value is close to 0.5, it represents the main storage mechanisms in the electrode is the intercalation-based behavior. If the *b* value is close to 1, it presents the capacitive effect playing a dominant role. After calculation shown in Figure 7d, the slopes (*b*_1_ and *b*_2_) by fitting peak 1 and peak 2 in the CV curves of CMLN were 0.52 and 0.64, which are near to 0.5, suggesting that the Li^+^ storage mechanism in CMLN anode is mainly controlled by the diffusion-controlled electrochemical process. The capacitive contribution of the CMLN electrode was further analyzed by fitting the areas of the CV curves as shown in Figure 7b. As shown in Figure 7c, the capacitive contribution of the CMLN electrode is 57% at a low sweep rate of 0.2 mV s^−1^. With the increasing of sweep rates, it can reach up to 87% at a high scan rate of 1.0 mV s^−1^. The high ratio of the capacitive contribution under the high current density is due to the fast kinetic process under the high charge–discharge rate. Additionally, the Li^+^ diffusion coefficient in the electrode can be obtained based on the following Equation (3) [42]:

Ip = 2.6 × 10^5^n^3/2^D^1/2^AC_o_v^1/2^ (3)

where Ip is the peak current in the CV curve, n is the number of the transferred electrons per species reaction, A is the surface area of the cathode, D is the diffusion coefficient of Li^+^, C_o_ is the concentration of Li^+^ in the electrode and v is the scan rate. The fitting line based on the relationship of Ip and the v^1/2^ based on peak 2 in the CV curves is shown in Figure 7e. The slope of the fitting line of the CMLN and CLN electrode was 0.473 and 1.035, respectively. After calculation based on Equation (3), the diffusion coefficient of Li^+^ (D) in the CMLN was about 6.51 × 10^−12^ cm^2^ s^−1^, which was higher than that of the CLN (1.14 × 10^−12^ cm^2^ s^−1^). The fast Li^+^ transfer in the designed CMLN electrode can be attributed to the fact that the abundant mesopores are helpful for the penetration of the electrolyte, and the N-doping can further promote the wettability of the materials with the electrolyte. The electrochemical impedance spectroscopy (EIS) analysis was further employed to evaluate the charge–transfer resistance (*R_ct_*) in the electrodes. The Nyquist plots (Figure 7f) consist of the semicircles in the high frequency region and an inclined line in the low frequency part. The semicircles correspond to the charge transfer resistance between electrode and electrolyte (*R_ct_*). It can be found from Figure 7f that CMLN shows *R_ct_* of 150 Ω, but the CLN displays the much higher *R_ct_* of 380 Ω, which suggested that electronic transfer rate in the battery with CMLN anode was much faster [43]. Above all, the fast Li^+^ diffusion and electronic transfer in the designed CMLN anode contributed to the excellent electrochemical performance. 

## 3. Materials and Methods

### 3.1. Materials Synthesis 

**Figure 8:**
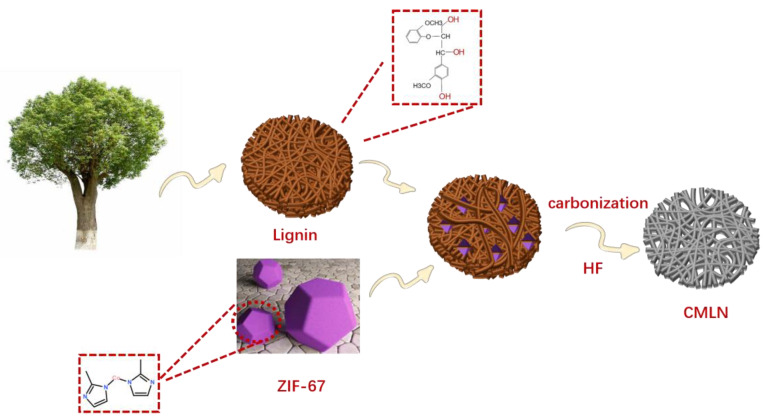
Fabrication process of the CMLN porous carbon.

The fabrication process was shown in Figure 8. Firstly, a total of 1.16 g of Co (NO_3_)_2_ 6H_2_O and 1.31 g of 2-methylimidazole were dissolved in 125 mL of methanol, respectively. Afterwards, the two solutions were mixed and stirred at room temperature for 30 min. The zeolitic imidazolate framework 67 (ZIF-67) precipitate was collected by centrifugation and then was washed followed by drying in an oven at 60 °C for 8 h.

Next, 3 g lignin and 0.3 g ZIF-67 were dispersed in 60 mL of deionized water with stirring. After that, the mixtures were added into a Teflon container followed by a hydrothermal reaction at 200 °C for 12 h. Next, the sample was collected by centrifugation and dried at 60 °C for 8 h. Finally, the sample was carbonized in a tube furnace at 750 °C for 3 h. After etching with HF acid to remove Co ions, the final composite was denoted as CMLN (carbonized MOF-based lignin). At the same time, a comparative experiment was carried out with pure lignin with the same preparation conditions. The reference sample was named as CLN (carbonized lignin).

### 3.2. Materials Characterization 

Scanning electron microscopy (SEM, JSM-7800F) and transmission electron microscopy (TEM, JEM-2100 (UHR), Philips, Tokyo, Japan) were used to observe the morphologies of the samples. Crystalline structure of the material was investigated with X-ray diffraction (XRD, XRD-6100, Shimadzu, Osaka, Japan) at a scan rate of 2° min^−1^ in the range of 10° to 80°. Thermogravimetric analyzer (TGA, TGA-Q500, Newcastle, TA, USA) was used to measure the content of the carbon in the sample. The character of the carbon in the composites was further investigated by Raman analysis with a Bruker Optics Senterra Labram Spectrometer (Bruker Optics, Saarbrucken, Germany). A BET analyzer (ASAP, Micromeritics, Atlanta, GA, USA) was used to analyze the pore size and the specific surface area. 

### 3.3. Electrochemical Measurements

The coin cells were assembled in the glove box filled with argon (Mikrouna, Shanghai, China). The anodes were made from a slurry mixture of active material (CMLN or CLN anode material), Super P and polyvinylidene fluoride (PVDF) adhesive in the weight ratio of 8:1:1. Copper foil was used as a collector and dried in a vacuum drying oven. Lithium sheets were used as counter electrodes for the cathode. The assembled cell (CR2016) was tested for charge–discharge characteristics using the battery system (Landt, Wuhan, China). Electrochemical impedance spectroscopy (EIS) and Cyclic voltammetry (CV) were conducted with an electrochemical workshop (CHI604E). 

## 4. Conclusions

In conclusion, we introduced a method for preparing mesoporous carbon materials with high surface area and porosity from the natural lignin assisted by MOF templates in this work. The obtained CMLN carbon composites show abundant mesopores (pore sizes distributed at 3.9 nm, 12.8 nm and 45.6 nm) and a high surface area of 288.7766 cm^2^ g^−1^. As an anode for LIBs, the CMLN displays a high specific capacity of 420 m Ah g^−1^ at 200 mA g^−1^ with the capacity retention of 99% after 300 cycles, which are better than those of most anodes reported in previous studies. The CMLN also displays outstanding long-cycle performance with the capacity retention of 85% after 1000 cycles, indicating the practical application value of a CMLN anode in LIBs. More importantly, the carbon source is mainly derived from wasted lignin, which can decrease the cost of the anode materials and reduce environment pollution. All in all, this work provided an effective and low-cost strategy for designing a porous carbon anode with high performance for LIBs or other secondary batteries.

## Figures and Tables

**Table 1:** A comparison of performance of porous carbon anodes reported in references.

Carbon	The Loading of Active Substances (mg cm^−2^)	Current Density (mA g^−1^)	Specific Capacity (mAh g^−1^)	Cycle Performance	Ref.
Spongy pomelo peels	0.5	90	452	98% 200 cycles	[31]
Mushroom	0.6	100	310	99% 450 cycles	[32]
Corn silk powders	0.8–1.0	200	210	99% 150 cycles	[33]
Graphite	1.5	100	100	93% 100 cycles	[34]
Loofah	1.0–2.0	100	250	90% 400 cycles	[35]
Sisal fiber	1.0	100	283	91% 30 cycles	[36]
Broad beans	2.0	372	262	87% 100 cycles	[37]
Sweet potato	1.8	100	320	98% 100 cycles	[38]
Bamboo wood	2.5–3.0	100	<300	95% 300 cycles	[39]
Coffee oil	2.0	100	274	99% 250 cycles	[40]
CMLN	1.5–2.0	200	420	99% 300 cycles	This work

## Data Availability

Not applicable.

## Supplementary Materials

The following supporting information can be downloaded at: https://www.mdpi.com/article/10.3390/ijms24010284/s1, Figure S1: CV curves of CLN at different scan rates from 0.2 to 1 mV s^−1^.
