# Supplementary material for: A Lignin-Based Carbon Anode with Long-Cycle Stability for Li-Ion Batteries
Source: Int J Mol Sci. 2022 Dec 23;24(1):284. doi: 10.3390/ijms24010284 (PMC9820563; doi:10.3390/ijms24010284)
Supplement: Supplementary file 1 [file ijms-24-00284-s001.zip › ijms-2067834-supplementary.pdf]

Supporting Information

A lignin-based carbon anode with long-cycle stability for Li-ion batteries

Shiyue Li[a], Wenbin Luo[a], Zhenyu Jiang[a], Jie Lu[a], Jian Du [a], Yehan Tao[a], Yi Cheng[a*], Haisong Wang[a*]

[a] *School of Light Industry and Chemical Engineering, Dalian Polytechnic University, Dalian, 116034, China*

**Corresponding author: Yi Cheng (chengyi18@dlpu.edu.cn); Haisong Wang (wanghs@dlpu.edu.cn)*

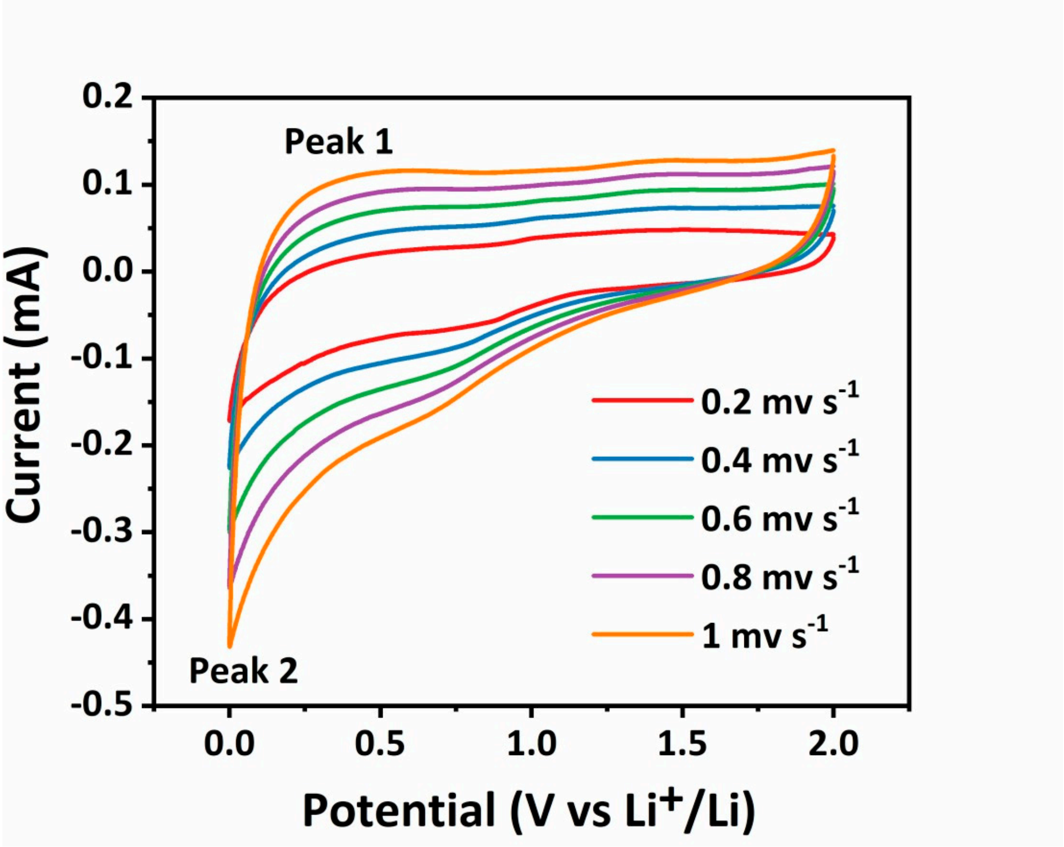

Figure S1. CV curves of CLN at different scan rates from 0.2 to 1 mV s^{-1};
